# Personalia

**Published:** 1913-10-04

**Authors:** 


### Personalia.

Sir Rickman Godlee, Bart., President of the Royal College of Surgeons, London, has accepted an invitation to confer the Fellowship of the American College of Surgeons at the first convocation of the institution, which will be held at Chicago, on November 13 next. It is stated that no fewer than 1,400 surgeons of the United States and Canada are to be created Fellows on this occasion.

The workmen of Sir W. G. Armstrong, Whitworth, and Co., Ltd., Elswick Works, Newcastle, have endowed a child's cot in the "Victoria" Ward of the Royal Victoria Infirmary, in memory of the late Mr. Thomas Buckham. The endowment is the result of a specially organised fund of £500, in addition to the usual weekly charitable contribution. The inscription on the tablet above the cot is as follows: "The 'Thomas Buckham Memorial Cot,' endowed by the workmen of Elswick Works, in memory of Thomas Buckham, who was for twenty-four years—1888 to 1912—one of their representatives on the House Committee of the Royal Victoria Infirmary."

